# Peptide Self-Assembly into Hydrogels for Biomedical Applications Related to Hydroxyapatite

**DOI:** 10.3390/gels5010014

**Published:** 2019-03-06

**Authors:** Manuel Rivas, Luís J. del Valle, Carlos Alemán, Jordi Puiggalí

**Affiliations:** 1 Chemical Engineering Department, Escola d’Enginyeria de Barcelona Est-EEBE, c/Eduard Maristany 10-14, 08019 Barcelona, Spain; manolorivas68@yahoo.es (M.R.); luis.javier.del.valle@upc.edu (L.J.d.V.); 2 Barcelona Research Center for Multiscale Science and Engineering, Universitat Politècnica de Catalunya, Escola d’Enginyeria de Barcelona Est-EEBE, c/Eduard Maristany 10-14, 08019 Barcelona, Spain

**Keywords:** amphiphilic peptides, hydrogels, hydroxyapatite, tissue regeneration, bone, teeth, drug release

## Abstract

Amphiphilic peptides can be self-assembled by establishing physical cross-links involving hydrogen bonds and electrostatic interactions with divalent ions. The derived hydrogels have promising properties due to their biocompatibility, reversibility, trigger capability, and tunability. Peptide hydrogels can mimic the extracellular matrix and favor the growth of hydroxyapatite (HAp) as well as its encapsulation. Newly designed materials offer great perspectives for applications in the regeneration of hard tissues such as bones, teeth, and cartilage. Furthermore, development of drug delivery systems based on HAp and peptide self-assembly is attracting attention.

## 1. Introduction

Peptide self-assembly allows new materials to be obtained through a bottom-up methodology [1]. Within this context, different building blocks based on peptides (i.e., from dipeptides to amphiphilic block copolymers) have been developed [2]. These systems have wide applications in the biomedical field (e.g., tissue, bone, and cartilage regeneration [3,4,5], or controlled drug release), being inspired from nature in multiple cases. Thus, self-assembly is characteristic of actin fibrils in eukaryotic cells, of the aggregation of fibrin in blood coagulation, and of amyloid fibrils, which can be employed as a component of biomembranes [6], biosensors [7], or hydrogels [8], in addition to being involved in well-known degenerative diseases (e.g., Alzheimer’s, Parkinson’s, and diabetes).

Multiple studies have been performed concerning the use of short peptide sequences as coatings, gels, and electroactive materials, as have recently been reviewed [9,10,11,12]. Self-assembled systems based on peptides offer clear advantages considering their biocompatibility, mechanical robustness, capability to be reversibly disassembled, and capability to tailor their functional behavior. The potential of self-assembled peptides is continuously increasing, and it is likely that the commercialization of these kinds of materials is approaching. Self-assembly can involve short sequences such as diphenyalanine [13,14], although single amino acids can also lead to self-aggregated fibrils [15].

Hydrogels are hydrophilic networks with a great capacity to retain water and have a great similarity with biological tissues. Hydrogels can be classified considering the type of cross-links (i.e., physical or chemical) and their ability to incorporate chemical agents and cells. Furthermore, these systems can be designed to render shape memory effects, stimuli-responsive smart materials, and in situ gelling polymers. These are ideal for tissue regeneration since they can be used as injectable materials able to adapt to the form of tissue cavities. Applications of hydrogels have been extensively reviewed [16,17,18], and it is clear that peptide-based hydrogelators have a great potential for biomedical applications due to their controllable self-assembly and biocompatibility, and their possibility to be formed under physiological conditions [19].

Hydroxyapatite (HAp) is a key component of hard tissues with recognized bioactivity, biocompatibility, and osteoconductivity. HAp has been extensively studied as an artificial bone substitute [20,21], in addition to the great efforts undertaken to develop bionanocomposites for applications in the biomedical field that varied from drug encapsulation to tissue engineering [22,23].

The present review focuses on the recent advances in hydrogels based on peptides and utilized in biomedical applications related to HAp. To this end, we begin with describing the self-assembly of peptides and HAp characteristics in Section 2 and Section 3, respectively. Next, HAp nanocomposites and hydrogels based on peptide self-assembly that are employed for biomedical applications are addressed in Section 4 and Section 5, respectively. Finally, specific applications related to nanoparticles/nanocapsules are discussed in Section 6 and materials employed for the regeneration of hard tissues, with the differentiation in advances concerning bones, teeth, and cartilage are accounted for in Section 7, followed by conclusions in Section 8.

## 2. Peptide Self-Assembly

At present, self-assembled peptides are ideal systems for the development of new biomaterials. Various advantages that can be mentioned are as follows [24]: (a) peptides with specific sequences can be easily obtained by solid-phase synthesis; (b) the small size of peptides allows the design of appropriate sequences for assembly into supramolecular structures; and (c) new peptides can be inspired from naturally self-assembling protein motifs. Therefore, self-assembled peptides (SAPs) can be based on weak specific interactions (i.e., hydrogen bonds and π–π stacking) or strong non-specific interactions (i.e., electrostatic), and α-helices, β-sheets, coiled-coils, or other naturally occurring motifs [25,26,27]. Peptides can also self-assemble into nanostructures through metal–ligand interactions. Metal coordination has become a powerful tool for constructing diverse molecular architectures and also for tuning the final assembly [28].

The self-assembly process is mainly driven by thermodynamics, but kinetic factors also have a critical relevance concerning structural modulation and function integration, as reviewed by Wang et al. [29]. The reverse process of assembly also has a significant role in the modulation and finalization of the activity of supramolecular complexes. Self-assembly and disassembly processes are ubiquitous in nature and can be exploited for biomedical applications such as therapeutics and diagnostics. Recent advances in the dynamic control of small molecules’ assemblies were reviewed by Feng et al. [30].

α-Helices comprise both hydrophilic and hydrophobic amino acid residues, and are characterized by a secondary structure defined by a periodicity of 3.6 amino acids per turn. These helical motifs can be self-assembled into coiled-coil conformations due to their amphiphilic character [31]. The main problem of such assemblies is the requirement of long amino acid sequences to stabilize the helical structure. Thus, the initial attempts involved sequences of 21–28 units, as reviewed in [32].

Collagen-mimetic peptides have a great applied interest since collagen plays a fundamental structural role in both tissues and extracellular matrix (ECM) [33]. Collagen is constituted by a supercoiling of three linear peptidic chains that gives rise to supramolecular fibrils. Amino acid sequences are mainly characterized by the presence of glycine, which avoids steric hindrances due to the lack of side groups; proline or hydroxyproline; and a third amino acid.

**Figure 1:**
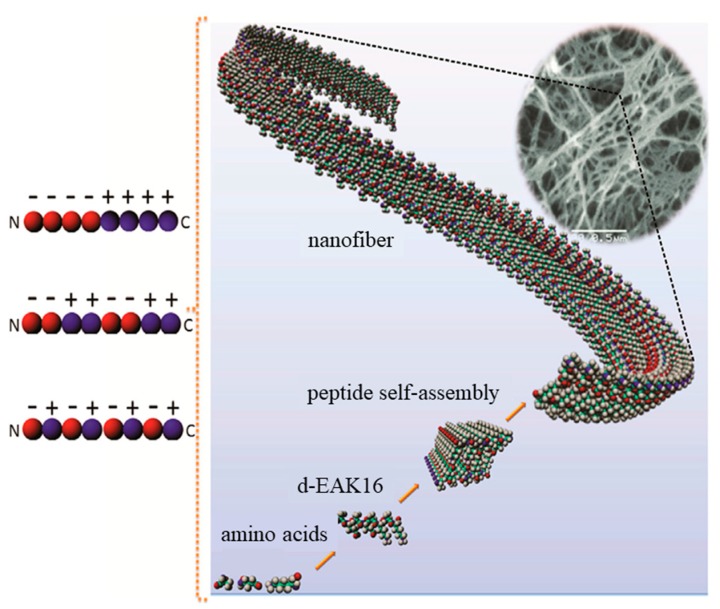
Self-assembly of a peptide consisting of alanine (A), glutamic acid (E), and lysine (K) (d-EAK) into nanofibers, which were then entangled into 3D scaffolds. The self-assembling process was a consequence of complementary ionic interactions between charged residues (red for negatively charged glutamic acid and blue for the positively charged lysine residues), which could have different arrangements: modulus I,− − − − + + + +; modulus II,− − + + − − + +; modulus III, − + − + − + − +). Adapted and reprinted with permission from [34,38].

β-Sheet peptides can be self-assembled into 3D-nanofibrous structures (e.g., hydrogels) when ionic self-complementary peptides are involved (Figure 1) [34]. Other self-assembly possibilities are based on the alternation of hydrophobic and hydrophilic residues into a β-sheet structure (e.g., peptides based on the Arg-Ala-Asp-Ala sequence named RADA-like SAPs), β-hairpin peptides, and β-sheet tapes [3,35,36]. These self-assembled structures render a great variety of morphologies such as nanofibers, nanotubes, nanovesicles, and nanoparticles [37].

Self-assembled peptides able to form β-structures can be classified according to five main architectures: (a) ionic self-complementary peptides; (b) peptides having an alternate disposition of hydrophilic and hydrophobic residues (i.e., RADA peptides); (c) l,d-heterochiral peptides, (d) peptide amphiphiles (PAs), and (e) *N*-protected peptides.

Ionic self-complementary peptides are based on the alternation of hydrophobic residues (e.g., alanine, isoleucine, or phenylalanine) and hydrophilic residues with positive (e.g., lysine or arginine) or negative (e.g., aspartic or glutamic acids) charges [34,39]. These complementary co-assembled peptides (CAPs) are consequently based on the interactions (attractive or repulsive) between peptides having opposite electric charges [40]. Nanofibrillar structures can be formed from molecular assemblies of positive Ac-(LKLH)_3_-CONH_2_ and negative (Ac-(LDLD)_3_CONH_2_ peptides. The derived β-sheets were thus constituted by double layers with hydrophobic and hydrophilic sides, the first ones being placed in inner pockets and the second ones exposed to aqueous environments. 

RADA and similar sequences (e.g., Ac-(RARADADA)_2_-CONH_2_ [41] and Ac-(RADA)_4_-CONH_2_ [42]) also form self-assembled structures in aqueous media where charged side groups are oriented on one side of the sheet and hydrophobic side groups are on the other side. The characteristic RADA sequence appears interesting to promote wound healing, cell culture, and synapse growth due to its great similarity with the RGD sequence that is characterized by its well-known cell adhesion properties. 

The functionalization of RADA peptides is a good strategy to control cell behaviors. Thus, scaffolds derived from RGD, DGR, and PRG-modified RAD16 peptides showed a clear improvement in cell attachment, spreading, migration, and osteogenic differentiation [43,44].

Alternation of l- and d-amino acids has been proved efficient to favor self-assembled structures. A simple sequence of three hydrophobic amino acids can lead to an amphiphilic arrangement with hydrophobic side chains and hydrophilic backbone groups [45]. Cyclic peptides constituted by alternating l,d-amino acids are able to self-assemble and form nanotubes [46]. Great attention is currently being given to the effect caused by the incorporation of unnatural d-amino acids in the self-assembling peptide motifs, as recently reviewed by Melchionna et al. [47]. 

**Figure 2:**
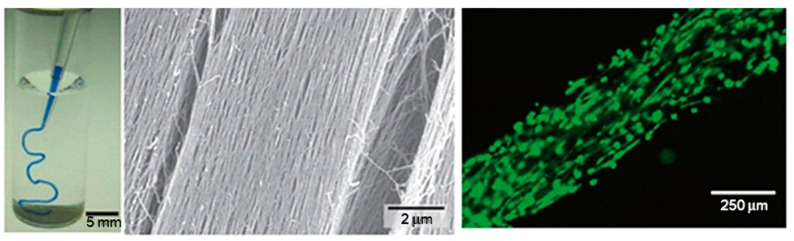
Formation of a noodle-like string from a peptide-amphiphile, colored with tryptan blue. (**left**) Pipetting was performed in a phosphate-buffered saline solution. (**middle**) SEM micrograph showing the highly aligned nanofiber bundles that constitutes the gel string. Gel strings can be used to encapsulate and align cells (e.g., human mesenchymal stem cells, hMSCs) along the axis of the gel (**right**). Adapted and reprinted with permission from [51].

The biological potential of fiber-forming peptide amphiphile molecules was revealed in 2001 [48]. The molecules were constituted by a hydrophobic tail linked to a hydrophilic amino acid sequence. Effective sequences were selected trying to favor β-sheet arrangements, to display cell adhesive properties (e.g., containing epitope Arg-Gly-Asp-Ser, RGDS, ligands [49,50]) and to promote mineralization by favoring HAp growth (e.g., containing phosphorylated serine residues able to attract calcium ions). Furthermore, thermal annealing processes were found to make feasible the formation of liquid crystalline solutions that rendered aligned domains under shearing forces. In the presence of divalent cations, these domains could result in gels with macroscopic fiber alignment (Figure 2) [51]. PA molecules are able to self-assemble in aqueous media into cylindrical nanofibers, as a result of both the establishment of hydrogen bonds between amide groups and the hydrophobic collapse of alkyl tails. Self-assembly can be induced by low pH and interactions with divalent ions. The latter option is more interesting for biomedical applications that require physiological pH.

Peptide self-assembly can be enhanced by the incorporation of appropriate *N*-terminal groups. The 9-fluorenylmethyloxycarbonyl (Fmoc) group is a good example due to the additional driving forces that can be established (e.g., hydrogen bonding from the carbonyl group, aromatic and hydrophobic interactions from the fluorenyl ring, and steric optimization from the methoxycarbonyl linker [52]. A variety of functional aromatic moieties (e.g., spiropyrans, stylbenes, carbazoles) have also been investigated and found to give a certain degree of functionality to the derived hydrogels [53]. 

Hydrogels based on fibrils based on the coassembly of Arg and Asp dipeptides (i.e., Fmoc-FR-NH_2_ and Fmoc-FN-OH) have been found effective to mimic the integrin-binding RGD peptide of fibronectin. The indicated amino acid residues had an orientation in the formed supramolecular fibrils that facilitated the promotion of cell growth. Interestingly, covalent connections between Arg and Asp motifs were avoided [54]. 

## 3. Hydroxyapatite

HAp is an inorganic component defined by the chemical formula Ca_10_(PO_4_)_6_(OH)_2_ that can be produced as nanocrystals by living systems under mild temperature and pressure conditions. HAp crystallizes in the monoclinic *P*2_1_/*b* space group (*a* = 0.984 nm, *b* = 2*a*, *c* = 0.688 nm and *γ* = 120º), but at temperatures above 250 °C can undergo a structural transition towards a hexagonal phase (*a* = *b* = 0.943 nm, *c* = 0.689 nm, and *γ* = 120º) [55,56]. This phase can be stabilized by the presence of impurities, like those derived from a partial substitution of hydroxide (e.g., by fluoride or chloride ions).

Biological HAp mainly forms part of animal bones, tendons, and teeth. At present, HAp can be considered one of the most-employed materials in hard tissue engineering due to three basic features: biocompatibility, bioactivity, and osteoconductivity [57]. Composite materials based on HAp can combine the advantages of each component (inorganic or organic) in order to get similar structures and properties to those found in nature. 

Considerable efforts are focused on the synthesis of nanohydroxyapatite (nHAp) particles (both in crystalline and amorphous forms). In this way, properties can be varied according to modifications in the composition and even morphology of nanoparticles [58,59]. The synthetic path is mainly performed according to two aqueous precipitation reactions [60]:

10 Ca(OH)_2_ + 6 H_3_PO_4_ → Ca_10_(PO_4_)_6_(OH)_2_ + 18 H_2_O, (1)

10 Ca(NO_3_)_2_ + 6 (NH_4_)_2_HPO_4_ + 2 H_2_O → Ca_10_(PO_4_)_6_(OH)_2_ + 12 NH_4_NO_3_ + 8 HNO_3_. (2)

**Figure 3:**
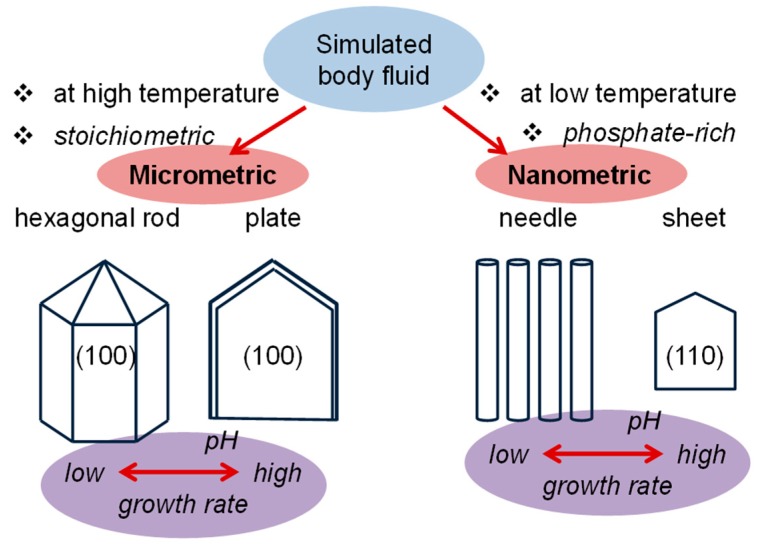
Change in the morphology of hydroxyapatite (HAp) crystals in simulated body fluid (BF) solutions according to stoichiometry, temperature, and pH. Growth rate becomes clearly enhanced by the increase of the pH of the medium. Indices indicate the crystal growth faces. Based on Reference [65].

Final characteristics are highly influenced by which method is used to mix the reactants and the speed at which they are mixed, pH conditions (Figure 3), and the incorporation of surfactants and chelating agents in the medium. Other processes, such as hydrothermal [61], sol–gel [62], sonochemical [63], and emulsification [64] have been proposed in order to increase the control of the crystal morphologies. 

The low cost, biocompatibility, and biodegradability of HAp justify its use in a wide range of applications. For example, HAp has been explored as a non-viral vector taking into account the facility of preparing HAp/DNA complexes that can be incorporated into cells and release DNA after dissolution of calcium phosphate in the low-pH acidic media of cell endosomal compartments [66,67,68]. Antibiotics such as chloramphenicol have also recently been encapsulated into HAp particles, giving rise to a proven selective antitumoral effect after their release inside cells [69].

## 4. Hydroxyapatite Nanocomposites

It has been postulated that mineralization in natural systems is initiated by the formation of poorly crystalline calcium apatite, which subsequently undergo phase transitions towards a stable HAp with higher crystallinity [70,71]. Anionic groups (i.e., those from acidic proteins of the ECM or from synthetic peptide-based hydrogels) are useful for binding inorganic calcium cations and aligning them in the growing crystal lattice [72]. There is a clear correspondence between the cell dimensions of the hexagonal phase of HAp and those of the repeat unit in β-sheet structures (i.e., the distances between two strands and between two residues are close to 0.48 nm and 0.69 nm, which correspond to half of the *a* and *b* axes and the *c* axis of HAp, respectively). In nature, the negatively charged surfaces of collagen fibrils nucleate the formation of the inorganic HAp phase [73]. 

In a similar way, synthetic PAs can be designed in order to self-assemble into nanofibers that promote HAp mineralization. Basically, the crystallographic *c* axis of the HAp hexagonal phase becomes oriented along the long axis of the peptide fiber. The ability to incorporate bioactive adhesion sequences (e.g., the RGD sequence) enhances the potential application of new synthetic hydrogel scaffolds for the regeneration of soft and hard tissues.

nHAp particles have a high surface area, and consequently a great proportion of ions become located at their surface. This feature has clear repercussions on the biological performance of the material, and specifically leads to an enhancement of cell adhesion and proliferation, osteointegration, and cell differentiation. In this way, the rapid growth of new tissues can be favored [74]. nHAp appears as an ideal component to obtain nanocomposite materials for biomedical applications, which are constituted by at least two different chemical phases and the corresponding interphase. Properties of nanocomposites can be strongly modified as a function of the characteristics of: (a) the filler (e.g., chemical constitution, intrinsic properties, morphology, size distribution, etc.); (b) the polymer matrix (e.g., molecular weight, mechanical and thermal properties, etc.); and (c) the polymer/filler mixture (e.g., ratio between components, interphase, degree of dispersion, etc.). The preparation of bionanocomposites also requires biocompatibility, nontoxicity, and significant degradation rates of both filler and matrix. Basically, a simple embedding of nHAp into a polymer matrix is enough to get a suitable nanocomposite arrangement. To this end, both thermo-mechanical (e.g., injection and extrusion [75,76]) and physico-chemical (e.g., coprecipitation and solvent casting) methods have been extensively applied.

The surface of nHAp is usually modified in order to improve the interphase characteristics with the organic matrix. In this way, surfactant molecules (e.g., oleic and stearic acids [77]) have been employed, and even grafting reactions (e.g., by ring-opening of lactide [78]) have been highly effective for both the compatibilization and improvement of colloidal stability in such a way that particle aggregation is avoided [79].

Collagen [80], gelatin [81,82], alginate [83], and chitosan [84] are typical natural polymers that have been considered with the aim of obtaining scaffolds incorporating nHAp for tissue regeneration. Modified natural polymers such as cellulose acetate [85] and synthetic polymers such as polylactide [86], poly(lactide-*co*-glycolide) [87], or polycaprolactone [88] have also been employed.

HAp composites can be prepared according different methodologies, being the most applied: (a) In situ coprecipitation of nHAp in a co-solution with the polymer selected as a matrix. This process can avoid typical agglomeration problems of other simple mechanical mixing processes. Even the crystallization of HAp can be delayed if some chelating compounds (e.g., polyacrylic acid for the calcium ions [89]) are added in the solution. (b) Dispersion of nHAp particles into the monomer (e.g., methacrylate anhydride [90]). In this case, particles become coated by a polymer shell (ex situ process) and aggregation problems are also diminished. (c) Electrospinning of polymer solutions incorporating nHAp particles. This is a promising process since it allows an easy preparation of fibrous and porous scaffolds with a biomimicking structure [91]. Finally, (d) self-assembly as a typical bioinspired process. For example, calcium and phosphate ions of the growing nHAp crystals can be assembled through ionic interactions with peptide functional groups (e.g., from collagen) [92,93]. 

In fact, around 200 acidic proteins have been proposed for biomimetic mineralization processes, which involved different functions such as inhibition, nucleation, or more usually to act as simple templates to favor the epitaxial growth of nanocrystals [94,95]. Silk [96], fibrinogen [97], and SAPs can form amyloid nanofibers and 3D hydrogels [98] able to bind calcium phosphates and favor the growth of nHAp [99].

**Figure 4:**
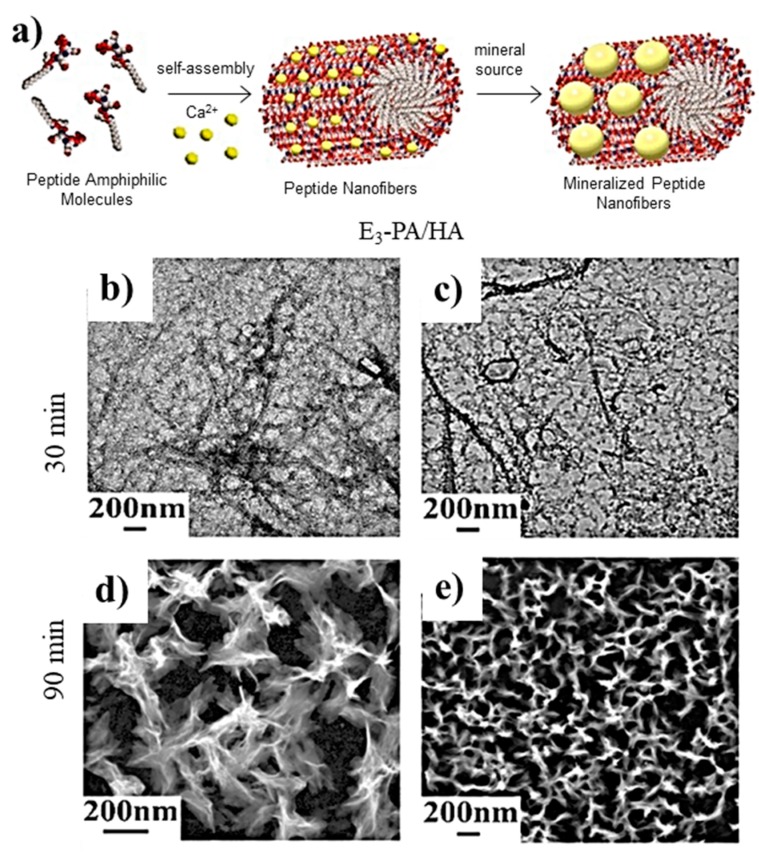
(**a**) Scheme showing the formation of nanofibers from peptide amphiphile molecules (PAs) and the subsequent mineralization. Micrographs taken after (**b**,**c**) 30 and (**d**,**e**) 90 min of calcium phosphate mineralization where nanofiber (**b**,**c**) and plate-like (**d**,**e**) morphologies are visible. Reproduced from [100].

PAs are ideal templates for the deposition of HAp crystals since they can mimic, for example, the phosphoserine-rich motifs of dentin proteins. Thus, an amphiphile having polar groups based on three glutamic acid (E) units (i.e., Lauryl-VVAGEEE (E3-PA)) has been found highly efficient for nucleating calcium phosphate and inducing the formation of HAp (Figure 4) [100].

PA nanofibers containing units of serine or phosphoserine (PA-S) were able to nucleate carbonated HAp spheroidal crystals when exposed to a calcium-supplemented medium. These fibers could also be mixed (up to a 5 wt%) with peptide amphiphiles containing the biological adhesion epitope RGDS. The mineralized nanofibers were found to promote the osteogenic differentiation of human mesenchymal stem cells (hMSCs) [101].

**Figure 5:**
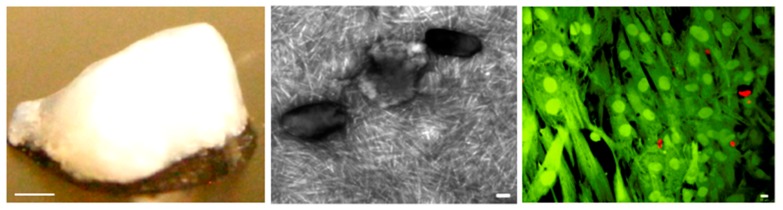
(**left**) Macroscopic, (**middle**) TEM, and (**right**) confocal images of PA-RGDS/PA-S (1:1) hydrogel with 50 wt% HAp. The right image shows encapsulated hMSCs, viable and dead cells appearing in fluorescent green and red colors, respectively. Scale bars correspond to 1 mm (**left**), 100 nm (**middle**), and 10 μm (**right**). Reproduced from [102].

Anderson et al. [102] prepared a biomimetic assembly consisting of PA nanofibers interspersed with nHAp. Specifically, a 1:1 mixture of a bioactive PA inscribed with the RGDS ligand to enhance osteoconductivity and a PA having an outer domain of inert serine with a strong gelating effect (PA-RGDS/PA-S (1:1)) was considered. The resulting scaffold was self-supporting, able to induce osteogenic differentiation, able to retain embedded HAp even at high concentration, and appeared interesting for bone tissue regeneration (Figure 5). The viscoelastic properties of the biphasic composite were found optimal for a HAp content of 50 wt%, an increase of the ratio between storage and loss moduli (as indicator of a predominant elastic character) from 4.1 to 8.5 being detected when nanoparticles were incorporated.

Multicomponent hydrogels based on HAp and different ratios of two peptide building blocks, FmocFF and FmocR, were developed to obtain 3D scaffolds with tunable properties. These materials showed a high potential for bone tissue engineering due to their good biocompatibility and high cell adhesion [103].

## 5. Hydrogels Based on Peptide Self-Assembly with Interest in Tissue Regeneration

Different synthetic hydrogels have been developed for tissue applications, making it possible to achieve a great control of physical properties. Polymethacrylate and polyethylene glycol have been extensively considered as synthetic polymers [71,104,105], but they have some problems for rendering an efficient encapsulation of cells under physiological conditions via self-assembly. Furthermore, the use of polymethyl methacrylate (PMMA) is limited due to the exothermic polymerization reaction involved in the in situ formation of the material and due also to its non-degradable nature that involves a risk of foreign body response and a delay of the healing process. 

Peptide hydrogels can achieve a high water content (i.e., up to 95 wt%) and thixotropic properties [106,107] since their formation is based on physical cross-links (e.g., hydrogen bonds, ion bonding, hydrophobic interactions). These links can be controlled according to the specific peptide sequence, peptide concentration, pH, and ionic force. Applications of these hydrogels include drug delivery vehicles [108] and cell-culturing materials [109,110] due to their great safety and affinity with cells [111,112]. Hydrogels can also be easily injected, and consequently they have a great potential as bone-filling materials.

Gels based on PAs offer great advantages, such as the easy production of self-assembled three-dimensional nanofibrous networks. These appear optimum to favor cell diffusion, cell responsive degradation, and biological signaling [113]. Mechanical properties similar to the natural ECM can be obtained without employing chemical cross-linking agents. In this way, reactions that are adverse for tissues can be avoided.

The self-assembly of peptides can be initiated by screening their charged groups, which can typically take place through pH changes or the addition of multivalent ions to the physiological medium [3,72]. Cylindrical nanostructures with peptide signals on their outer periphery are usually derived. The formation of non-covalent cross-links between fibers renders the final macroscopic hydrogel [114,115]. The peptide sequence can be modified in order to enhance specific tissue priorities (e.g., osteogenic differentiation, drug delivery, or tunable gelation) [116,117,118]. 

**Figure 6:**
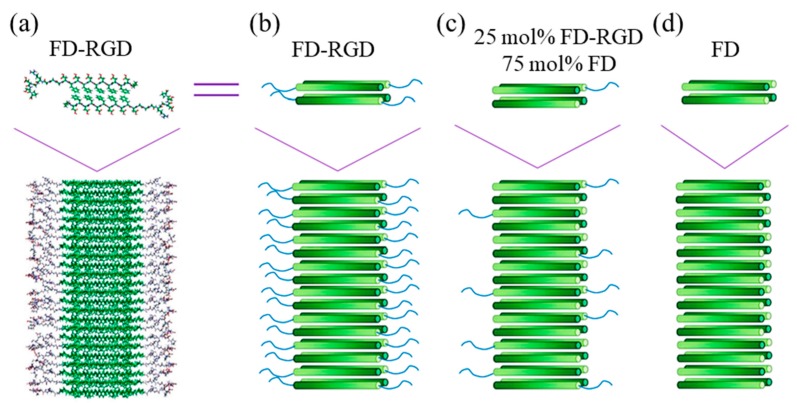
(**a**) Scheme showing a molecular model of Pro-D**FDFDFDFDFD**GGGRGDS-Pro (FD-RGD) assemblies) and (**b**–**d**) cylinder representations with a gradient color from the N- to C-termini (dark to bright, respectively). The β-strand conformation positions the hydrophobic Phe side chains from both layers facing each other, while the hydrophilic side chains point to the surrounding aqueous phase. The top schemes show four peptides arranged in a bilayer that constitutes the fibril shown in the bottom scheme. Reproduced from [120].

Pro-D**FDFDFDFDFD**-Pro is a clear example of an amphiphilic and anionic peptide that can form hydrogels by self-assembling into β-sheets. Its capacity to induce biomineralization is an added value for bone regeneration [119]. This peptide has recently been linked to the integrin RGD motif, giving rise to Pro-D**FDFDFDFDFD**GGGRGDS-Pro (FD-RGD)—a peptide that could be combined up to a 25 mol% with FD without disrupting the self-assembling fibril structure and increasing the density of osteoblasts cultured in the corresponding hydrogels [118]. In fact, FD-RGD was specifically designed to facilitate the juxtaposition with FD by satisfying cross-strand hydrogen bonds in the mixed fibrils (Figure 6). Thus, twelve residues were identical to those characteristic of FD, and three Gly amino acids were the linker with the integrin motif, which protruded out of the fibril to the solvent phase. 

Note that small peptides can be incorporated into a polymer matrix in order to guide the biomineralization process. Genetically engineered peptides that selectively bind to inorganic compounds (GEPIs) have been developed by exposing a pool of random amino acid sequences (placed on the surface of a bacteriophage or bacteria host organism) to a target inorganic substrate [121,122]. The more effective binding sequences have subsequently been identified [123] to produce GEPIs able to enhance the mineral formation [124,125,126].

**Figure 7:**
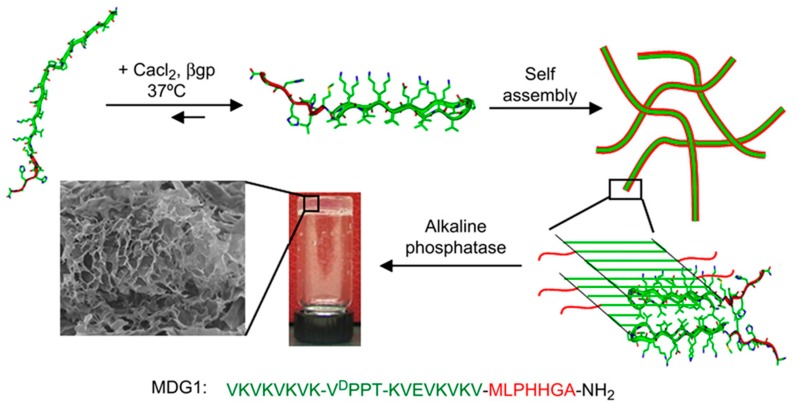
Scheme showing the self-assembly of MDG1 and the formation of three-dimensional gels after mineralization. Reproduced from [121].

Gungormous et al. [121] designed a 27-residue peptide (MDG1) able to undergo triggered folding to form an unsymmetrical β-hairpin. A mechanical rigid and self-supporting hydrogel was obtained as a consequence of the self-assembly induced by the increase of the solution ionic strength. Basically, an unfolded conformation was initially expected due to the unfavorable electrostatic interactions between residue side chains, but the peptide became able to fold after charges were screened by the addition of CaCl_2_ and β-glycerolphosphate (β-GP) (Figure 7). The 20 C-terminal residues were chosen for their ability to self-assemble and consisted of two β-strands with an alternating disposition of hydrophobic and hydrophilic residues. These two sequences were connected by an intermediate residue sequence able to render a β-turn. The N-terminal was constituted by a heptapeptide able to initiate the mineralization process of calcium and phosphate to produce HAp.

## 6. Nanoparticles and Nanocapsules Based on Peptide Self-Assembly

**Figure 8:**
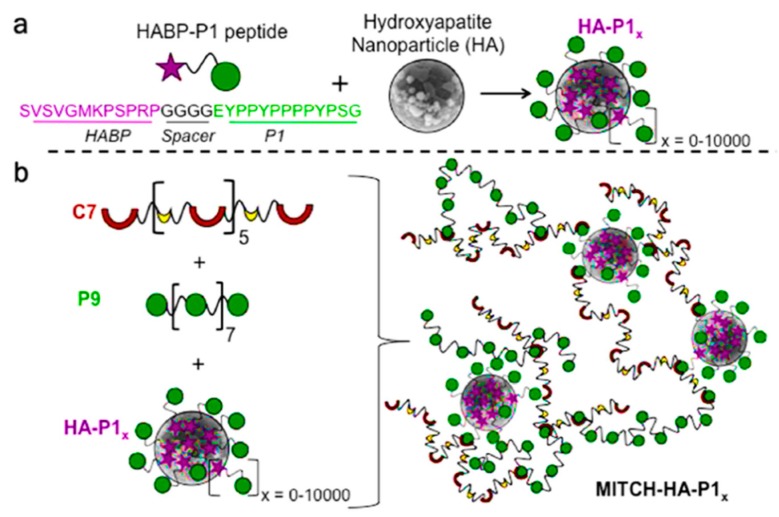
Schematic representation of a polymer–nanoparticle hydrogel with specific molecular recognition. (**a**) Binding of a number *x* of selected HABP-P1 peptides to the surface of hydroxyapatite nanoparticles. (**b**) Mixing of HA-P1x with recombinant proteins P9 and C7 induces the formation of a supramolecular hydrogel with direct physical linkages between the inorganic and organic phases. Adapted and reprinted with permission from [130].

Polymer–nanoparticle (PNP) materials constitute a new class of hydrogels that are formed through nonspecific interactions between polymer chains and the surfaces of colloidal particles [127,128,129]. Nanoparticles can be coated with a bifunctional peptide that can establish physical cross-links with a recombinantly engineered protein. This molecular recognition allows a control over the number of physical cross-links and thereby of the mechanical (e.g., stiffness) performance of the material. It has been demonstrated that this approach is interesting for the therapeutic delivery of cells for bone regeneration [130]. Thus, nHAp particles were embedded within a protein-engineered hydrogel (Figure 8) though specific physical interactions that could be established using a designed peptide. This hydrogel can act as a stem-cell carrier after the corresponding encapsulation and promote bone regeneration when implanted into defect sites. 

**Figure 9:**
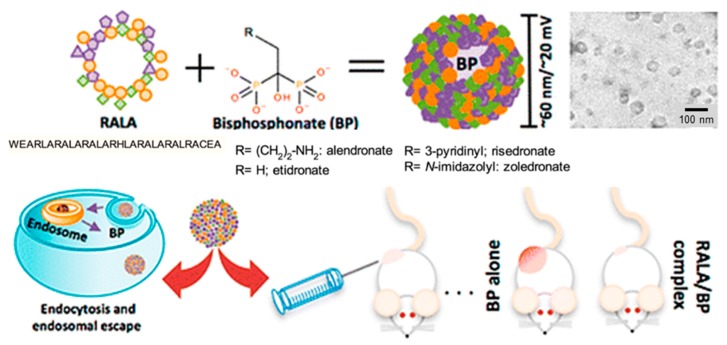
Nanoparticles with antitumoral properties based on self-assembled RALA peptides coated with BPs. Reproduced from [129].

Cationic nanoparticles can easily be prepared from amphiphilic peptides, and have been found highly effective as non-viral gene delivery vehicles to transport anionic nucleic acids into different cell types [129]. Nanoparticles based on peptides rich in arginine and having the RALA sequence were proved to be non-cytotoxic and capable of crossing the plasma membrane of cancer cells. Nanoparticles with diameters lower than 100 nm and stable over a large range of temperatures for more than 6 h were prepared by incubating RALA peptides with alendronate or other related nitrogen-containing bisphosphonates (BPs). These compounds can establish good electrostatic interactions to efficiently coat the peptide-based nanocapsule. The derived particles had pH responsiveness due to conformational changes. Specifically, the peptide adopted an alpha-helical conformation that could be disrupted in the acidic medium of the endosome, facilitating the release of loaded BP into the cytosol (Figure 9) [131]. 

Selected BPs have antitumor activity and inhibited tumor growth, migration, invasion, adhesion, and angiogenesis [132,133]. BPs having hydroxyl and two phosphonate groups flanking a carbon atom showed a great affinity with HAp bone matrix, and consequently the circulating particles could be retained, avoiding a fast excretion by the kidneys.

## 7. Hard Tissue Regeneration

The maintenance of healthy bones, cartilage, and teeth is becoming one of the most relevant problems for an aging society, due also to intrinsic difficulties associated with the capability of old people to recover from fractures. 

Three components (i.e., cells, scaffolds, and signals) must be considered in regenerative medicine [134,135]. Concerning cells, bone marrow-derived hMSCs have been extensively considered due to their high capability to be differentiated into osteoblasts [136,137]. However, their proliferative activity tends to decrease with age [138], and consequently other alternatives have been considered (e.g., dental pulp stem cells (DPSCs)) [139].

Hard tissue engineering has some inherent problems due to the limited number of cells that can be seeded effectively in the scaffold and also their uneven distribution that reduces the activity of incorporated cells in osteogenesis repair [140]. Consequently, efforts have been focused on promoting the accumulation of fibrous proteins to increase the area for the adhesion of seeded cells. In this case, problems related to the inhibition of nutrient delivery and vascularization have been reported [141].

Basically, new formulated bionanocomposite systems try to mimic the characteristics of the extracellular matrix: (a) a hybrid structure of inorganic matter (e.g., HAp) and organic macromolecules (e.g., polysaccharides and proteins); (b) a morphology based on particles with a high aspect ratio and diameter in the nanoscale range. 

Effective nutrient delivery is an important factor for tissue repair due to the strong metabolic demand of osteoblastic cells during tissue regeneration [141,142]. In general, cells tend to grow preferentially on the outer scaffold regions [143], being dynamic culture systems developed to avoid nutrient transport limitations in static culture. Bokhari et al. [142] used dynamic cell seeding and culturing techniques, demonstrating a higher penetration of cells (up to 3 mm) in HAp-modified scaffolds coated with RAD16-I. The peptide enhanced osteoblast differentiation and provided an appropriate environment for osteoblast growth. 

**Figure 10:**
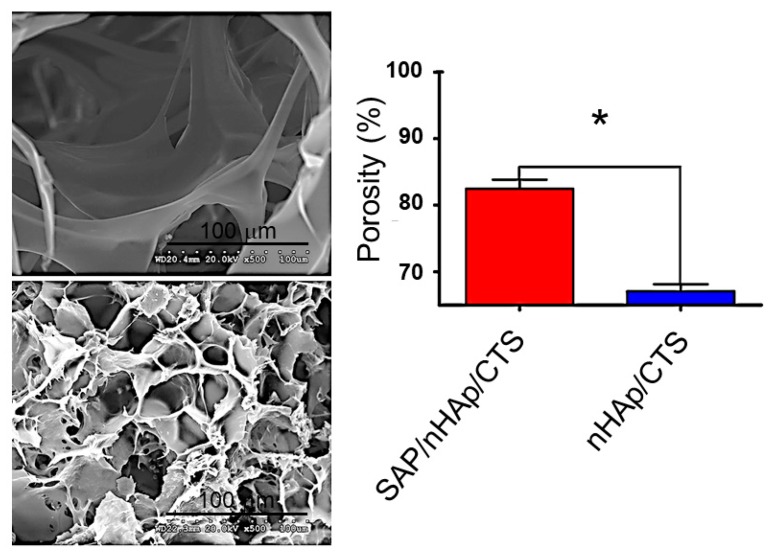
The morphology and SEM of (**top left**) nHAp/ CTS and (**bottom left**) SAP/nHAp/CTS. (**right**) The porosity of the SAP/nHAp/CTS was 84.53%, which was significantly higher than that of nHAp/CTS, 67.97%, * *p* < 0.05. Reproduced from [144]. CTS: chitosan; SAP: self-assembled peptides.

Different porous scaffolds based on nHAp and chitosan (CTS) have been considered for tissue engineering applications. Nevertheless, some factors are not highly positive, the poor cell adhesion likely being the main problem limiting the material for an appropriate cell seeding. An interesting solution was the incorporation of a commercialized SAP hydrogel consisting of standard amino acids (1% *w*/*v*) and water that could have a similar behavior to the extracellular matrix. Specifically, Zhu et al. [144] demonstrated that SAP/nHA/CTS scaffolds increased the adhesion of bone mesenchymal stem cells and enhanced the mechanical properties of the scaffold. Assays were successfully focused on the repair of a femoral condylar bone defect in a mouse model. Experimental results indicated that healing could be achieved after 12 weeks. Figure 10 compares the geometry of SAP/nHA/CTS and nHA/CTS scaffolds, with larger apertures and lesser porosity being detected for scaffolds without SAP. It was postulated that these peptides were able to fulfill the large pores and subsequently self-assembled. 

The osteogenic differentiation of MSCs in three-dimensional scaffolds based on RADA SAPs has been evaluated with promising results. Thus, high alkaline phosphatase activity and osteocalcin (OC) contents were determined, with the detection of a clear growth of a mineralized extracellular matrix within the hydrogel. Mesenchymal stem cells were able to undergo a 3D differentiation to form mineralized matrices within the scaffold hydrogel [145].

**Figure 11:**
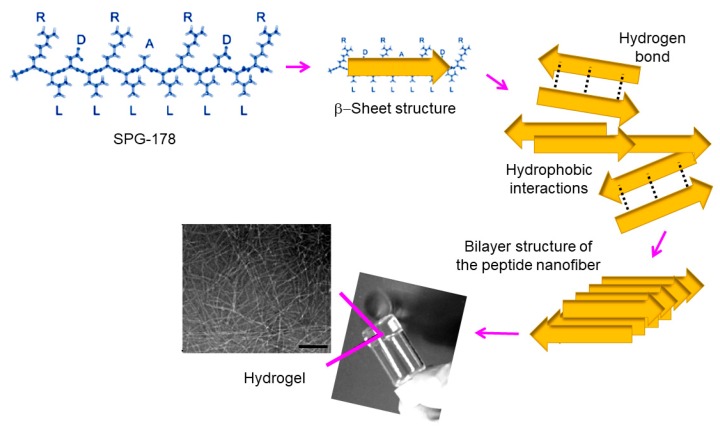
Chemical structure of the self-assembled SPG-178 peptide and scheme showing the corresponding hydrogel formation. Scale bar = 100 nm. Scheme based on [135].

Several self-assembled RADA peptides (e.g., RAD16-I and RAD16-II characterized by RADARADARADARADA (modulusI) and RARADADARARADADA (modulus II) sequences, respectively) which easily form hydrogel scaffolds have been employed in tissue engineering [107,146,147]. However, the presence of carboxylic groups leads to low pH levels that are harmful for cells and host tissues. Therefore, neutralization procedures have employed [148,149]. The use of non-acidic self-assembled peptides can also solve this problem, with peptides based on arginine, alanine, leucine, and aspartic acid (e.g., SPG-178 peptide with a RLDLRLALRLDLR sequence) being interesting in this case. Specifically, SPG-18 [110,150] has an isoelectric point of 11.5 and can form a stable hydrogel at neutral pH (Figure 11). It has been demonstrated that DPSC proliferation and osteogenic differentiation were successful using the SPG-718 hydrogel and an osteogenic induction medium containing recombinant human bone morphogenetic protein-4 (rhBMP-4). Gene expression levels of osteopontin, osteocalcin, and collagen type I were observed to increase significantly under these conditions [135]. 

**Figure 12:**
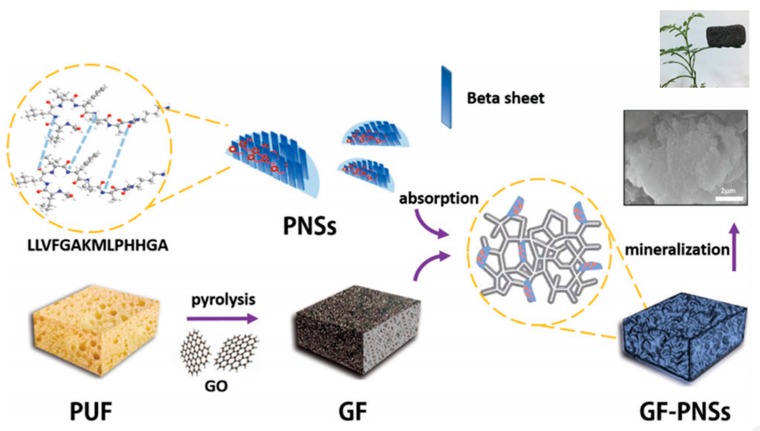
Biomimetic fabrication of 3D graphene (GF)-PNSs-HAp scaffold. PUF is a polyurethane foam used as a template for GF that can subsequently be removed from the flame, and GO is graphene oxide. Reproduced from [96]. PNS: peptide nanosheet.

Li et al. [95] prepared new hybrid materials based on peptide nanosheets (PNSs) derived from the self-assembly of LLVFGAKMLPHHGA and their noncovalent conjugation onto a graphene (GF) support (Figure 12). This 3D hybrid scaffold was suitable for the growth of HAp, giving rise to materials having adjustable shape, very low weight (0.017 g cm^−3^), high porosity (5.17 m^2^ g^−1^) that allows vascularization and transport of nutrients, excellent biocompatibility and, consequently, high potential for bone tissue and biomedical applications.

**Figure 13:**
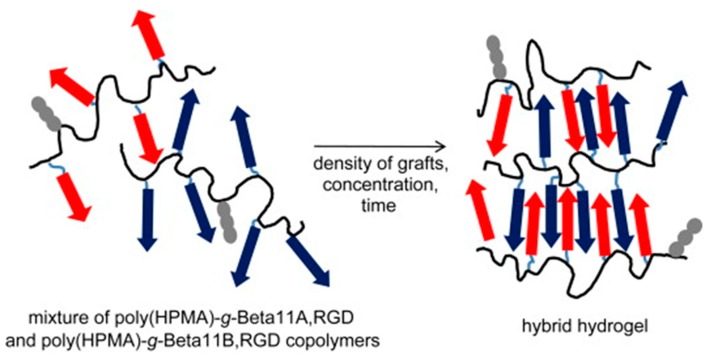
Hybrid hydrogel derived from the self-assembly of poly(HPMA)-*g*-*β*-sheet complementary copolymers (blue and red arrows). Grafted RGD motifs are indicated by gray fragments. Reproduced from [151]. HPMA: *N*-(2-hydroxypropyl)methacrylamide.

Complex systems (Figure 13) based on *N*-(2-hydroxypropyl)methacrylamide (HPMA) copolymers grafted with two complementary β-sheet peptides (i.e., Beta11A (Ac–TTRFTTTFTTT–amide) and Beta11B (Ac–TTEFTTTFETT–amide)) and a RGD motif (i.e., NH_2_–GGRGDSP–amide) have also been designed for tissue regeneration [151]. Self-assembled fibrils were effective in orienting the growth of HAp, giving rise to a good control of mineralization. Furthermore, the system overcame some disadvantages of collagen-based scaffolds such as batch-to-batch variation, immunogenicity, complex molecular structure, and poor mechanical strength [152]. Moreover, copolymers had a good solubility, which appears to be advantageous with respect to scaffolds based only on β-sheet peptides.

In vivo assays using different types of peptide amphiphiles were carried out by Mata et al. [152]. A strong ability to promote the nucleation of HAp crystals when phosphoserine units (S(P)) were incorporated into the peptide sequence was demonstrated. It is known from in vitro assays that non-collagenous proteins rich in S(P) residues favor mineral nucleation, stimulate gene expression, and enhance the osteoblast differentiation of MSCs [153]. The RGDS fibronectin epitope was also incorporated to favor the adhesion of cells involved in bone regeneration (e.g., mesenchymal stem cells, osteoprogenitor cells, osteoblasts, and vascular tissue cells).

Polymer–HAp nanocomposites were prepared using thermoreversible self-assembled polymer templates (i.e., pentablock copolymers based on Pluronic F127 (poly(ethylene oxide)-*b*-poly(propylene oxide)-*b*-poly(ethylene oxide)) and anionic and zwitterionic blocks) with attached HAp-nucleating peptides (i.e., DSKSDSSKSESDSS) at each end. These systems allowed a good control of mineralization and showed a liquid-to-solid transition at physiological temperatures. Thus, nanocomposites could be injectable, able to conform to the shape of bone or cartilage defects, and form solids at physiological conditions [154]. 

### 7.1. Bone Regeneration

Bone tissue engineering can overcome the problems inherent to the transplantation of allogenic bone grafts (e.g., long-term chronic pain, nerve injury, risk of new fractures, immunogenic rejection, or even disease transmission [155,156]), providing new potential treatments for the repair of bone defects [157]. Ideal bone implants should be: (a) osteoconductive, having a capacity to favor the attachment, survival, migration, and distribution of cells; (b) osteoinductive, responding to external stimuli to enhance the attachment, survival, migration, and distribution of cells; and c) osteogenic, containing stem and osteogenic cells for regeneration [158]. 

**Figure 14:**
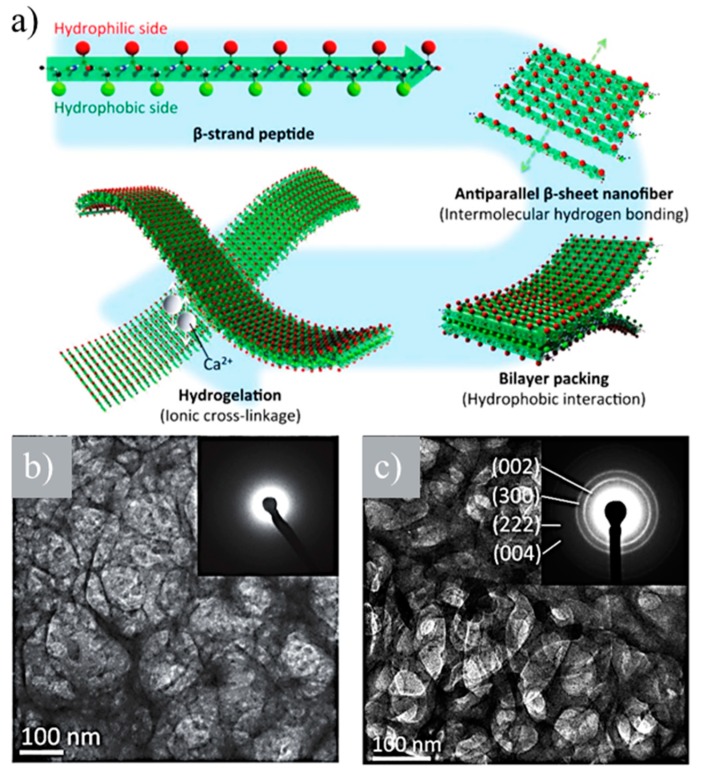
(**a**) β-strand (LE)_8_ and (VEVSVKVS)_2_ peptides formed antiparallel β-sheets, which packed face-to-face together through hydrophobic interactions between the two hydrophobic sides. The hydrophilic surfaces of bilayer nanofibers were subsequently able to cross-link by the ionic interaction between the anionic carboxyl group and the cationic calcium ion. (**b**) TEM image and amorphous calcium phosphate (ACP) amorphous electron diffraction pattern (inset) of the network derived from calcium phosphate and (LE)_8_ peptide. (**c**) TEM image and HAp crystalline electron diffraction pattern (inset) of the network derived from calcium phosphate and (VEVSVKVS)_2_ peptide. Reproduced from [98].

Two novel bone-filling materials were developed using (LE)_8_ and (VEVSVKVS)_2_ β-sheet-forming peptides [98]. These were based on the alternating disposition of hydrophobic (i.e., leucine (L) or valine (V)) and hydrophilic (i.e., glutamic acid (E), serine (S), or lysine (K)) units. Both peptides were able to self-assemble, giving rise to nanofibers that formed hydrogels in the presence of calcium ions (Figure 14a). An ionic cross-linkage was established between carboxyl groups of the glutamic acid side chains of nanofibers and added calcium ions. (VEVSVKVS)_2_ was able to retain the hydrogel structure at higher percentages of calcium ions (i.e., >1.0 × 10^−2^ M) than (LE)_8_ due to its lower number of glutamic acid residues per molecule that prevents collapse. Viscoelasticity increased with calcium ion concentration, and it was possible to obtain an appropriate strength for a bone-filling material. Amorphous calcium phosphate (ACP) and HAp were mineralized along peptide nanofibers of (LE)_8_ and (VEVSVKVS)_2_ under neutral and basic pHs, respectively (Figure 14b,c).

The enhancement of the scaffold bioactivity for bone regeneration can be achieved through the encapsulation of bioactive molecules (e.g., bone morphogenic proteins, BMPs). However, the release of these bio-factors is generally too fast to match the bone repair process, which requires several months to be completed [159,160]. Therefore, biomimetic peptide hydrogels based on BMPs are highly interesting since they may provide a sustained release of bioactive molecules through degradation during the whole bone regeneration process. Obviously, bioactive units can also be generated inside the gel in order to promote cell differentiation and osteogenesis [161,162]. 

Efforts have recently been focused on the development of peptides related to proteins having high osteoinductive properties, as it is the case of bone morphogenic protein-2 (BMP-2). In this way, problems related to its large size, insolubility, instability, high cost, and easy denaturation can be avoided. Thus, the bone morphogenetic protein-2 biomimetic peptide (BMPBP) has been proposed considering its reduced size (i.e., between 20 and 30 amino acids) and the presence of typical SSPVT morphogenic sequences. Scaffolds encapsulating BMPBP were able to induce osteogenic differentiation and promote bone tissue regeneration [163,164]. However, these scaffolds could not provide sufficient bioactivity to meet the requirements of clinical application. Quan et al. developed new hydrogels based on a BMPBP core containing 16 amino acids, phosphoserine as template to favor the deposition of calcium ions, an RGDS cell adhesion peptide, and polyaspartic acid to synergistically promote osteogenesis [165]. New scaffolds were found to significantly accelerate the in vivo formation of rat cranial bone when rat MSCs were also included.

### 7.2. Tooth Regeneration

Great efforts are currently focused on the prevention of dental caries. These mainly involve actions to avoid the formation of microbial films and the enhancement of the effective remineralization process of the initial dental decay [166,167]. 

Enamel is the hardest mineralized tissue of the human body and constitutes the external coating of teeth. This highly organized mineral is produced in the ectoderm germ layer and lacks collagen or other precursor proteins [168,169]. The high mechanical properties are the consequence of a hierarchic organization where thousands of HAp crystals are anisotropically arranged in packed bundles or rods (i.e., the *c*-axis of HAp becomes aligned with the rod long axis). Ameloblast cells produce matrix proteins such as amelogenin, which self-assemble into nanospheres [170] and regulate the oriented crystal growth of HAp and ameloblastin. The latter protein facilitates the demarcation of rod boundaries and promotes cell–matrix interactions [171]. Proteins are eliminated during the last step of enamel development by the action of proteolytic enzymes [172]. 

The use of liquids and pastes that contain nHAp for the repair of tooth surfaces is highly extended for remineralization of submicrometer-sized enamel lesions [166]. However, the treatment of larger cavities is more problematic and requires more complex solutions. Dental enamel is a hard biological tissue that cannot be repaired under the acid media derived from caries activity. The organic construction collapses, giving rise to the formation of a cavity. Self-assembled organic scaffolds are currently considered to control the crystal growth of nHAp and give rise to an ordered structure that mimics enamel. Representative advances on the development of self-assembled structures [50,173,174,175,176,177] appropriated for biomimetic enamel repair have been well summarized by Elkassas et al. [165].

Huang et al. [168] developed a bioactive matrix by self-assembly to induce the in vivo ectopic formation of enamel. A well-organized hierarchical structure of HAp crystals was derived in close resemblance with native enamel. The matrix was based on a branched peptide amphiphile bearing a high density of the epitope RGDS.

Self-assembled peptides (i.e., biomimetic P11-4 having the Ac-Gln-Gln-Arg-Phe-Glu-Trp-Glu-Phe-Glu-Gln-Gln-NH_2_ sequence [177]) have also been employed for caries treatment. Specifically, P11-4 is able to form a 3-D structure with Ca^+2^ binding sites, which can act as nucleation points for HAp. After the application of P11-4 on the tooth surface, the peptide self-assembles due to the low pH in the lesion and provides a scaffold similar to the enamel matrix [178]. 

**Figure 15:**
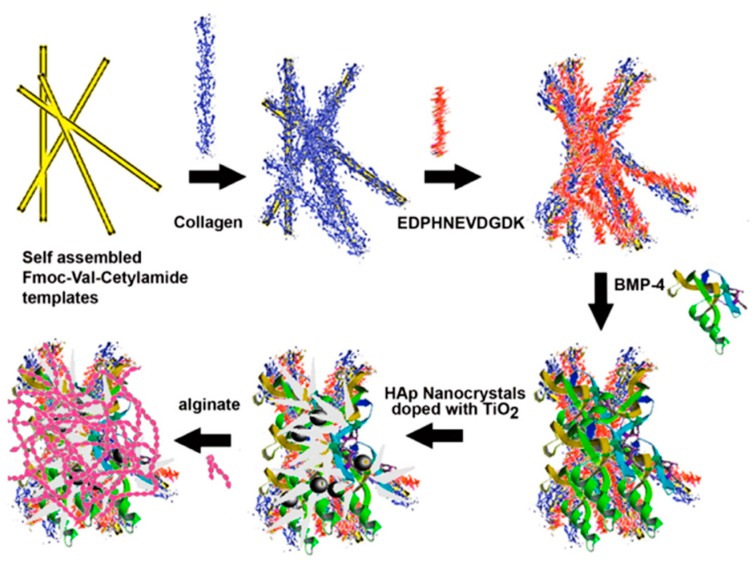
Scheme showing the preparation of 9-fluorenylmethyloxycarbonyl (Fmoc)-Val-cetylamide-collagen-DT–BMP-4 biocomposites by layer-by-layer (LBL) assembly. The assemblies were finally coated with 1% alginate to form scaffolds for bone tissue regeneration. Reproduced from [179].

Romanelli et al. [179] used Fmoc-Val-cetylamide to form self-assembled nanofibrous gels, which had great affinity towards HAp and served as templates to bound proteins that mimicked the ECM of osteoblasts. Layer-by-layer (LBL) assembly allowed the incorporation of collagen, the sialophosphoprotein (EDPHNEVDGDK) sequence from dentin, and the osteoinductive growth factor BMP-4. Assemblies were incubated with HAp nanocrystals, blended with varying mass percentages of TiO_2_, and finally coated with alginate to form three-dimensional scaffolds (Figure 15). These new materials were biodegradable and displayed a clear antibacterial activity. The ratio of TiO_2_ nanoparticles had a great impact on antibacterial (e.g., against *Staphylococcus aureus*) and biodegradability properties. Scaffolds were found to induce osteoblast differentiation and to proliferate and attach to mouse preosteoblast MC3T3-E1 cells.

Resin-based composites are usually employed for dental restoration, but the in vivo degradation of the adhesive–dentin interface is a recurrent problem that limits the lifetime of the clinical restoration [180]. Viable solutions are focused on providing a minimum integrity to the indicated interphase by means of a peptide-mediated remineralization of dentin [181]. The use of specific peptides with affinity for both HAp and collagen appear ideal to promote the remineralization of dentin and seal dentine tubules [182].

### 7.3. Cartilage Regeneration

Articular cartilage has a poor regenerative capacity, which is the main cause of the osteoarthritis disease typical of highly developed countries [183]. This consists of a depletion of glycosaminoglycans that causes a loss of mechanical properties and function in vitro [184]. 

Cartilage-to-cartilage integration is more complex than typical bone-to-bone integration due to the presence of stem cells and vascularity. The mechanical performance of cartilage depends on some characteristics that contribute negatively to healing and integrating with tissues [185]. Materials with suitable mechanical properties combine networks of collagen and glycosaminoglycans, which make cell migration and adhesion difficult [186]. Furthermore, the avascular character of cartilages disrupt their accessibility to nutrients and progenitor cells [187]. All these difficulties justify the great efforts that are currently focused on the achievement of a good biomimetic cartilage graft fixation and integration [188,189]. 

Chondrocytes with a well-known low metabolic activity are main agents responsible for cartilage repair. The implantation of undifferentiated MSCs has a limited interest since, although a certain capability to promote the accumulation of cartilage-like tissue has been detected, the results are not sufficiently satisfactory to render a reliable regeneration of articular cartilage [190]. The induction of chondrogenesis is a basic point when MSCs are expected to be employed for cartilage regeneration. The ex vivo chondrogenic preconditioning in defined culture medium able to promote differentiation and secretion of ECM [191] is a possibility for enhancing the regeneration potential, but at the same time the use of injectable hydrogels becomes precluded [192]. Interesting works have recently been performed seeding MSCs into a self-assembled peptide (AcN-(KLDL)_3_-CNH_2_) hydrogel able to support the chondrogenesis of the encapsulated MSCs [193,194] and facilitating the recovery of individual cell suspensions that is suited for injectable therapies.

A recent strategy consisted of the use of an osteochondral construct obtained from the interdigitation of HAp ceramic-based material with a functionally viable neocartilage prepared by self-assembly [189]. Glycosaminoglycan and collagen were the main components of the self-assembled neocartilage.

Suitable scaffolds for cartilage repair consist of three-dimensional templates in which attached or seeded chondrocytes produce and deposit a continuous ECM network. A great variety of natural and synthetic polymers are being considered for cartilage repair (e.g., collagen, alginate, polyglycolide, polylactide, polyethylene oxide [195,196,197,198]). Hydrogels derived from SAPs have also been proposed as appropriate environments for the retention of chondrocyte phenotype and the achievement of a cartilage extracellular matrix [199].

SAPs based on tryptophan and phenylalanine residues, which are placed on the middle and on the same side of the peptide to drive ribbon formation, and charged amino acids that favor and antiparallel sheet arrangement have been proposed [200]. Specifically, CH_3_COQQRFEWEFEQQNH_2_ (P_11_-4) CH_3_COQQRFOWOFEQQNH_2_ (P_11_-8) and CH_3_COSSRFOWOFESSNH_2_ (P_11_-12) sequences have been evaluated. These SAPs can be mixed with chondroitin sulfate and initially delivered as a non-viscous fluid that can be triggered to self-assemble once located in place. Hydrogels showed promising properties for biomedical applications in glycosaminoglycan-depleted tissues. The best results were achieved with P_11_-8 and P_11_-8 samples.

## 8. Conclusions

The number of works on the development of peptide-based hydrogels has increased exponentially since the discovery of the self-assembly capacity of molecules consisting of small peptide sequences. These hydrogelators have a wide range of applications, but the most promising ones concern the biomedical field, mainly as a consequence of the capacity of such materials to mimic the ECM. Properties like biocompatibility, establishment of physical and reversible cross-links, feasibility of cross-linking under physiological conditions, tunability, and trigger capacity justify the great interest in such materials. 

The research carried out so far highlights the diversity of appropriate sequences to induce self-assembly and the possibility of changing both structure and functionality. The great potential of such materials is obviously linked to the great variability in the composition and length of peptides, which opens the possibility of a tailored design to suit a specific property. 

The incorporation of charged amino acids in the peptide sequence promotes the interaction with calcium divalent ions, making it possible to use the derived hydrogels for the nucleation of HAp and even the development of nanocomposites. Promising results have been achieved in recent years concerning the development of hydrogels for the regeneration of hard tissues—mainly bone, teeth, and cartilage, as evidenced in the present review. The achievements attained in this field are very relevant, as it is expected that they will be able to reach an effective commercialization of these materials. The ability to gel under the action of suitable stimuli also allows the use of self-assembling peptides as injectable materials that can be fitted to the shape of the defect to be regenerated.

Different challenges must still be overcome in order to obtain a maximum benefit and attain an effective commercialization. Progress is needed for the development of smart materials susceptible to external stimuli (e.g., electrical signals, pH changes, addition of bone morphogenetic proteins) and especially for drug delivery systems based on self-assembling peptides and HAp.
